# Editorial: Canine osteosarcoma as a model in comparative oncology: Advances and perspective

**DOI:** 10.3389/fvets.2023.1141666

**Published:** 2023-01-31

**Authors:** Mariarita Romanucci, Raffaella De Maria, Emanuela Maria Morello, Leonardo Della Salda

**Affiliations:** ^1^Department of Veterinary Medicine, University of Teramo, Teramo, Italy; ^2^Department of Veterinary Sciences, University of Turin, Turin, Italy

**Keywords:** comparative oncology, dog, model, osteosarcoma, spontaneous tumors

Canine osteosarcoma (OSA) is an aggressive malignancy, sharing biological and clinical similarities with the human counterpart. The prognosis of patients with high-grade OSA still remains relatively poor in both species, with survival rates not having significantly improved during the recent decades. Thus, novel biomarkers of disease progression and response to treatment, as well as molecular targets for development of novel therapeutics, are urgently needed to improve the outcome of both human and canine OSA. Given the similarities between human and canine OSA, and the higher incidence rates of OSA in dogs, the canine population is a valid natural model of human disease. Therefore, the identification of specific altered pathways in canine OSA could facilitate the establishment of improved treatment strategies and provide the basis for the development of a personalized approach to OSA therapy in comparative oncology.

In this respect, the present Research Topic features original studies and reviews relevant to our theme of “*Canine osteosarcoma as a model in comparative oncology: Advances and perspective*” by bringing together scientific contributions from multiple experts in this field of study.

By an integrated analysis of whole-exome and RNA sequencing, the original research of Gola et al. provided the molecular characterization of a large number of canine OSA cell lines, allowing future investigations on their functional implications and drug response, and representing excellent translational models. In fact, cell lines constitute one of the most suitable and reproducible pre-clinical models and therefore, the knowledge of their molecular network is essential to explore oncogenic mechanisms and drug response (1). In particular, mutations in eight genes, previously described as human OSA drivers and including TP53, PTCH1, MED12, and PI3KCA, were detected in the investigated cell lines (Gola et al.).

MicroRNAs (miRNAs) are small non-coding RNAs involved in the regulation of gene expression, and a growing body of literature exists exploring the significance of their expression changes in OSA (2–10). miRNAs are also attractive molecules for biomarker/target discovery efforts (11–13). In this respect, Dailey et al. successfully identified miRNA expression changes associated with patient outcome in both canine OSA tumors and patient serum samples. Focusing on tumor-derived miRNAs associated with poor outcome, pathway and miRNA target prediction analyses were used to integrate miRNA and gene expression data to identify potential aberrant pathways contributing to OSA progression. These integrated analyses suggested that the interaction between OSA cells and the primary tumor microenvironment may contribute to the metastatic phenotype of aggressive tumors.

The importance of glucose transporter member 1 (GLUT-1, also known as SLC2A1), matrix metallopeptidase 3 (MMP3) and nuclear factor erythroid 2–related factor 2 (NFE2L2/NRF2) is also well-established in human OSA (14–17). For this purpose, Rutland et al. investigated the immunohistochemical expression of these cancer promoting proteins, that have been shown to be upregulated at the gene level in canine OSA compared to normal bone tissue (18). The study of Rutland et al. confirmed the expression of GLUT1, MMP3 and NRF2 in canine OSA, suggesting them as good potential candidates for prognostication and therapeutic targets, and encouraging clinical trials using drugs targeting these proteins.

Studies have also demonstrated the roles of parathyroid hormone-related protein (PTHrP) and its receptor (PTHR1) in the development, progression and metastasis of several tumors, including OSA. In this respect, the review of Al-Khan et al. highlighted the latest findings about functions of PTHrP and PTHR1 in normal and neoplastic tissues by focusing on their roles in OSA progression and discussing the possible related pathways in humans and canines.

Vasculogenic mimicry (VM) is a unique property of malignant cancer cells to create their own fluid-conducting microvascular channels without the involvement of endothelial cells, and has emerged as a potential target for anti-tumor therapy (19, 20). For this reason, the review of Massimini et al. illustrated the main findings concerning VM process in human OSA, as well as the related current knowledge in canine pathology and oncology, in order to provide a basis for future investigations on VM in canine tumors.

As well, in order to accelerate the understanding of the molecular basis of OSA, potentially facilitating a more rapid identification of novel therapeutic targets relevant to both people and dogs, the review of Simpson et al. focused on the shared molecular mechanisms between human and canine OSA, also presenting key differences revealed in comparative studies.

Evidence also suggests that OSA is an immunogenic tumor, and development of immunotherapies for the treatment of pulmonary micrometastases might improve long-term outcomes. The core hypothesis of adoptive natural killer (NK) cell therapy is the existence of a natural defect in innate immunity that can be restored by adoptive transfer of NK cells in cancer patients (21). In this respect, the perspective article of Kisseberth and Lee described the rationale for adoptive NK cell immunotherapy, NK cell biology, TGFβ and the immunosuppressive microenvironment in canine OSA, also illustrating the manufacturing of *ex vivo* expanded canine NK cells and providing perspectives on the present and future clinical applications of adoptive NK cell immunotherapy in spontaneous OSA and other tumors in dogs. The review of Razmara et al. also focused on the recent literature characterizing NK and T cell infiltration in OSA tumors and their prognostic significance in humans and dogs.

Finally, in the study of Flesner et al., a multimodal pain assessment methodology was used to evaluate pain relief after therapeutic intervention in dogs with primary bone cancer, suggesting that an improved assessment of pain severity and relief in dogs with cancer may allow a better evaluation of the efficacy of therapy. A direct benefit for people with cancer-induced bone pain was also highlighted, by potentially decreasing the amount of subtherapeutic novel drugs entering human clinical trials.

In conclusion, the studies collected in this Research Topic further support spontaneous OSA in dogs as a valuable model system to inform the development of new prognostic and therapeutic tools for both human and canine OSA. We hope that the contributing articles will inspire and encourage future studies on OSA pathogenesis, disease progression and therapeutic management in comparative oncology.

## Author contributions

All authors listed have made a substantial, direct, and intellectual contribution to the work and approved it for publication.
